# Genomic Sequence of *Streptococcus salivarius* MDI13 and *Latilactobacillus sakei* MEI5: Two Promising Probiotic Strains Isolated from European Hakes (*Merluccius merluccius*, L.)

**DOI:** 10.3390/vetsci11080365

**Published:** 2024-08-10

**Authors:** Lara Díaz-Formoso, Diogo Contente, Javier Feito, Pablo E. Hernández, Juan Borrero, Estefanía Muñoz-Atienza, Luis M. Cintas

**Affiliations:** Grupo de Seguridad y Calidad de los Alimentos por Bacterias Lácticas, Bacteriocinas y Probióticos (Grupo SEGABALBP), Sección Departamental de Nutrición y Ciencia de los Alimentos (Nutrición, Bromatología, Higiene y Seguridad Alimentaria), Facultad de Veterinaria, Universidad Complutense de Madrid, Avda. Puerta de Hierro, s/n, 28040 Madrid, Spain; lardia01@ucm.es (L.D.-F.); diogodas@ucm.es (D.C.); ehernan@ucm.es (P.E.H.); jborrero@ucm.es (J.B.); lcintas@ucm.es (L.M.C.)

**Keywords:** marine fish, probiotics, lactic acid bacteria (LAB), whole-genome sequencing (WGS), antimicrobial peptides

## Abstract

**Simple Summary:**

In fish farming, diseases have commonly been fought with the abusive use of antibiotics, which have caused antibiotic (multi)resistances in bacteria. Consequently, it is necessary to explore safe and environmentally friendly alternative approaches to improve the fish health and avoid the treatment of bacterial diseases with antibiotics such as the use of probiotics. This study focuses on bioinformatic and functional analyses of the genome sequences of *Streptococcus salivarius* MDI13 and *Latilactobacillus sakei* MEI5, two Lactic Acid Bacteria (LAB) isolated from the gut of European hakes (*Merluccius merluccius*, L.), a highly valued marine fish for Spanish gastronomy. The potential probiotic characteristics of both bacteria, and the lack of antibiotic resistance genes and virulence were confirmed. In addition, genes encoding three antimicrobial peptides (known as bacteriocins) were identified in the genome of *S. salivarius* MDI13. One of these in vitro-synthesized bacteriocins (BlpK) showed antimicrobial activity against two fish pathogens (namely *Lactococcus garvieae* and *Streptococcus parauberis*). Altogether, our results suggest that *S. salivarius* MDI13 and *L. sakei* MEI5 have a strong potential as probiotics to prevent bacterial diseases in fish farming.

**Abstract:**

Frequently, diseases in aquaculture have been fought indiscriminately with the use of antibiotics, which has led to the development and dissemination of (multiple) antibiotic resistances in bacteria. Consequently, it is necessary to look for alternative and complementary approaches to chemotheraphy that are safe for humans, animals, and the environment, such as the use of probiotics in fish farming. The objective of this work was the Whole-Genome Sequencing (WGS) and bioinformatic and functional analyses of *S. salivarius* MDI13 and *L. sakei* MEI5, two LAB strains isolated from the gut of commercial European hakes (*M. merluccius*, L.) caught in the Northeast Atlantic Ocean. The WGS and bioinformatic and functional analyses confirmed the lack of transferable antibiotic resistance genes, the lack of virulence and pathogenicity issues, and their potentially probiotic characteristics. Specifically, genes involved in adhesion and aggregation, vitamin biosynthesis, and amino acid metabolism were detected in both strains. In addition, genes related to lactic acid production, active metabolism, and/or adaptation to stress and adverse conditions in the host gastrointestinal tract were detected in *L. sakei* MEI5. Moreover, a gene cluster encoding three bacteriocins (SlvV, BlpK, and BlpE) was identified in the genome of *S. salivarius* MDI13. The in vitro-synthesized bacteriocin BlpK showed antimicrobial activity against the ichthyopathogens *Lc. garvieae* and *S. parauberis*. Altogether, our results suggest that *S. salivarius* MDI13 and *L. sakei* MEI5 have a strong potential as probiotics to prevent fish diseases in aquaculture as an appropriate alternative/complementary strategy to the use of antibiotics.

## 1. Introduction

Aquaculture is defined by the Food and Agriculture Organization of the United Nations (FAO) as the farming of aquatic animals, including fish, crustaceans, mollusks, and aquatic plants. In 2022, global aquatic animal production was approximately 223.2 million tons, reaching a historic record. Compared to 2020, aquaculture increased by 6.6%. Of the total production of aquatic animals, 89% was intended for human consumption, making them one of the most traded food products globally. Today, the challenge of feeding a growing population without depleting natural resources continues to grow. In this sense, aquatic food systems have great potential to meet the nutritious food needs of humanity [1]. One of the main issues in fish farming is the fish stress, which can be minimized by using the appropriate husbandry methods. However, stress can never be completely eliminated, which increases the susceptibility to disease occurrence and leads to massive mortalities. Bacteria are the primary pathogens affecting marine species, accounting for 54.9% of all infections [2]. Common pathogenic bacteria responsible of severe infections in numerous economically valuable fish species include *Vibrio* spp. (e.g., *Vibrio harveyi*, *Vibrio vulnificus*, *Vibrio parahaemolyticus*, and *Vibrio alginolyticus)*, *Listonella anguillarum*, *Tenacibacullum maritimum*, *Aeromonas salmonicida*, *Streptococcus parauberis*, and *Lactococcus garvieae*, amongst others. For example, diseases such as tenacibaculosis (caused by *T. maritimum*), which affects many marine fish such as turbot and sea bass, can have very high mortality rates. There is also vibriosis, which can cause the death of 50% of infected fish and shellfish. In addition, *Aeromonas* infections, such as *A. salmonicida*, are quite common and can be lethal, especially in cold-water salmonids. Regarding Gram-positive bacteria, *S. parauberis* is the main bacterial agent that causes a significant economic loss to the mariculture industry, and *Lc. garvieae* is a common agent that can provoke significant economic losses not only in several fresh fish species but also in saltwater species [2,3]. Traditionally, diseases in aquaculture have been fought with the indiscriminate use of antibiotics, which has led to the development and spread of (multiple) antibiotic resistances in bacteria. For this reason, it is necessary to search for new alternative/complementary strategies safe for humans, animals, and the environment, such as the use of probiotics in fish farming [4,5].

Probiotics are defined by the World Health Organization (WHO) and FAO as live microorganisms that, when supplied in sufficient quantity, enhance the health of the host [6]. In a broader definition, probiotics for aquaculture are considered as live microbial cultures that exert a beneficial effect on the host by (i) modifying its microbiota and/or that of its environment; (ii) enhancing the efficiency of feed assimilation and/or its nutritional value; (iii) improving the host response to disease; and/or (iv) increasing the quality of the aquatic environment in which the host grows [7,8,9].

Currently, only one microorganism is internationally authorized for use as a zootechnical additive in the aquaculture of all fish and crustacean species in the European Union (EU), namely *Pediococcus acidilactici* CNCM I-42622 (formerly MA18/5M), a strain registered under the trade name Bactocell^®^ [10]. Microorganisms proposed as probiotics in fish farming must display antimicrobial activity and must be considered safe not only for aquatic animals, but also for the surrounding environment and humans [11]. Most of the studies conducted in the field of probiotics for aquaculture focus on Lactic Acid Bacteria (LAB), awarded the Generally Recognized As Safe (GRAS) status by the Food and Drug Administration (FDA) and included in the Qualified Presumption of Safety (QPS) list established by the European Food Safety Authority (EFSA) due to their universal presence in food and their contribution to the healthy microbiota of the human gut [12,13,14,15,16]. LAB are the most studied group of microorganisms as probiotics for aquaculture and there are many studies evaluating their application in fish production. The main mechanisms by which probiotics can exert benefits in aquaculture include the following: (i) the modulation of the host microbiota and/or its environment; (ii) the improvement of feed utilization and/or its nutritional value; (iii) the reduction of host susceptibility to diseases; (iv) the improvement of host response to stress; (v) the enhancement of reprouctive function; and/or (vi) the improvement of water characteristics [9,17,18,19,20,21].

In general, in the process of the selection and evaluation of probiotic strains, their full and comprehensive characterization is essential to demonstrate their safety and beneficial effects on the host health. Recently, the EFSA has included Whole-Genome Sequencing (WGS) as a requisite for the characterization of bacteria and yeasts proposed as products or as enzyme-producing strains in food and feed [22]. Therefore, the study of the complete genome of probiotic strains, using Next-Generation Sequencing (NGS) techniques, has great relevance in the detection of genes responsible for their probiotic characteristics (e.g., production of antimicrobial compounds) and in the evaluation of their safety by studying the presence of antibiotic resistance and other virulence factor genes [23]. Among the desirable antimicrobial compounds for probiotic strains are bacteriocins, which are defined as ribosomally synthesized peptides produced and secreted by Gram-positive and Gram-negative bacteria that have a variable antimicrobial spectrum against bacterial pathogens [23].

Therefore, the overall objective of this work was the WGS, bioinformatic, and functional analyses and safety characterization of two strains (*S. salivarius* MDI13 and *L. sakei* MEI5) isolated from the gut of European hakes (*Merluccius merluccius*, L.) from the Northeast Atlantic Ocean for their potential application as probiotics in aquaculture.

## 2. Materials and Methods

### 2.1. Growth Conditions and Isolation of Genomic DNA

*S. salivarius* MDI13 and *L. sakei* MEI5 were isolated from non-eviscerated commercial European hakes (*Merluccius merluccius*, L.) caught from the Northeast Atlantic Ocean (Southwest of Ireland), provided by a Galician skipper dedicated to professional fishing. Both strains were grown in Man, Rogosa and Sharpe (MRS) agar (1.5%, *w*/*v*) plates (Oxoid, Basingstoke, UK) at 30 °C overnight.

The genomic DNA of the two strains was first extracted and purified using the DNeasy Blood & Tissue kit (Qiagen, Hilden, Alemania) following the manufacturer’s instruction, and then quantified (ng/µL) in a Qubit 4 fluorometer (Invitrogen, Waltham, MA, USA).

### 2.2. Whole-Genome Sequencing (WGS), Assembly, and Mapping

The WGS of both strains was carried out at the SeqCenter (Pittsburgh, PA, USA). In brief, the Illumina DNA Prep kit and 10 bp unique dual index (UDI) from Integrated DNA Technologies (IDT) were used to prepare libraries. Afterward, they were sequenced on an Illumina NextSeq 2000 (Illumina, San Diego, CA, USA), producing 2 × 150 bp reads. The BCL Convert software v3.9.3 (Illumina) was used to perform the demultiplexing, quality control, and adapter trimming. The Unicycler v0.4.8 program was used to assemble the resulting sequence reads into contigs [24]. Rounds of assembly polishing were conducted with the Pilon program (Oxford Nanopore Technologies, Oxford, UK). Assembly annotation was performed with the Rapid Annotations using Subsystems Technology (RAST) server [25]. In addition, genome mapping of the two strains was created by the Proksee web server (https://proksee.ca/ accessed on 6 August 2024) [26].

### 2.3. Bioinformatic (In Silico) Analyses

#### 2.3.1. Identification of Bacterial Species

Although the two probiotic candidates were formerly identified by DNA sequencing of the gene that encodes the 16S rRNA subunit (*16S rDNA*) as *S. salivarius* and *L. sakei*, their identification was validated by two different databases. Specifically, *SpeciesFinder v.2.0*., which identifies bacteria according to the complete sequence of the *16S rDNA*, and *KmerFinder v.3.0.2.*, which relies on the frequency of overlapping kmers (i.e., 16-mers) [27,28].

#### 2.3.2. Assembly Evaluation, Bacteriocin Identification, and Safety Evaluation

The assembled genomic sequences of the two strains were analyzed individually to confirm their taxonomic identification at the species level and to (i) evaluate the quality of the assembly; (ii) identify the genes and operons related to the biosynthesis and production of bacteriocins; and (iii) to evaluate their safety by identifying genes encoding potential virulence factors and antibiotic resistance genes by means of several web server programs (Table 1). The *RAST* platform was used to obtain general data for the two genomes analyzed and to assess the quality of the assembly by evaluating the L50 and N50 values. The identification of Clustered Regularly Interspaced Short Palindromic Repeats (CRISPRs) and CRISPR-associated proteins (*cas*) (CRISPR-Cas) systems, and mobile genetic elements, for instance active prophages, insertion sequences (ISs), and plasmids, was carried out using several bioinformatics pipelines (Table 1). Additionally, a phylogenetic WGS-based mapping and the genomic maps of the two strains were created by the Type (Strain) Genome Server (*TYGS*) and the *Proksee* web server, respectively (Table 1).

### 2.4. Evaluation of the Deconjugation of Bile Salts

The capacity of the two strains to deconjugate taurocholate and taurodeoxycholate was evaluated using a protocol described by Noriega et al. (2006) [40]. First, bacteria were inoculated in MRS broth (Oxoid) and incubated at 30 °C under aerobic conditions for 16 h. After incubation, 10 μL of the cultures was inoculated onto MRS agar (1.5%, *w*/*v*) plates supplemented with L-cysteine (0.05%, *w*/*v*, Merck, Darmstadt, Germany) and the respective bile salts (taurocholate or taurodeoxycholate, 0.5%, *w*/*v*, Sigma-Aldrich, Darmstadt, Germany), and incubated at 37 °C in anaerobiosis (AnaeroGen, Oxoid) for 72 h. A fresh fecal slurry from a healthy adult cow was employed as a positive control.

### 2.5. Evaluation of the Degradation of Mucin

The capacity of the two strains to degrade gastric mucin was evaluated following the method described by Zhou et al. (2001) [41]. Mucin from porcine stomach type III (Sigma-Aldrich) and agar were added into medium B without glucose at concentrations of 0.5 and 1.5% (*w*/*v*), respectively. In brief, 10 μL of cultures grown in MRS broth was spotted onto the medium B containing mucin. After the incubation at 37 °C for 72 h in anaerobiosis (AnaeroGen, Oxoid), the plates were stained with a 0.1% (*w*/*v*) solution of amido black (Merck KGaA) in 3.5 mol/L acetic acid for 30 min and then washed with 1.2 mol/L acetic acid (Merck KGaA). The appearance of a discolored zone around the bacterial spots indicated a positive result. A fresh fecal slurry from a healthy adult cow served as a positive control.

### 2.6. Evaluation of the Production of Biogenic Amine

The presence of *hdc*, *tdc*, *odc*, and *ldc*, encoding histidine decarboxylase (HDC), tyrosine decarboxylase (TDC), ornithine decarboxylase (ODC), and lysine decarboxylase (LDC), respectively, was evaluated by PCR. PCR amplifications were performed using previously reported primers [42,43,44,45] and the PCR products were visualized in an agarose (1,5% *w*/*v*) (Pronadisa, Madrid, Spain) gel with GelRed Nucleic Acid Gel Stain (Biotium, Inc., Fremont, CA, USA) using a ChemiDoc Imaging System (Bio-Rad Laboratories, Inc., Hercules, CA, USA).

### 2.7. Synthesis In Vitro of Bacteriocins and Evaluation of Their Antimicrobial Activity

Total genomic DNA from *S. salivarius* MDI13 was used to amplify the nucleotide sequences of the mature bacteriocins of interest (*slvV, blpK*, and *blpE*). Oligonucleotides (Table 2) were acquired from Thermo Fisher Scientific (Waltham, MA, USA). The nucleotide sequence of the T7 promoter was included in the forward primers, which was followed by the first 29, 28, and 27 bases corresponding to the genes of interest (*slvV, blpK*, and *blpE*, respectively) and starting with ATG. In the case of reverse primers, these contained the nucleotide sequence of the T7 terminator followed by the last 22, 24, and 24 bases of the gene of interest (*slvV*, *blpK*, and *blpE*, respectively). PCR amplifications were performed using the Phusion Hot Start II High-Fidelity DNA Polymerase (Thermo Fisher Scientific) and genomic DNA (5–100 ng). After visualization of PCR-amplified gene fragments as described above, amplicons were purified using the NucleoSpin^®^ Gel and PCR Clean-up kit (Macherey-Nagel™, Allentown, PA, USA), and quantified with a Qubit fluorometer system (Invitrogen, Waltham, MA, USA). To conduct in vitro cell-free protein synthesis (IV-CFPS) reactions, purified PCR amplicons were used as templates using the PURExpress In vitro Protein Synthesis Kit (New England Biolabs, Ipswich, MA, USA) and a final DNA concentration of 10 ng/µL, and then incubated at 37 °C for 2 h [46,47,48].

The antimicrobial activity of the in vitro-synthesized peptides (bacteriocins) was assessed using the Spot-On-Agar Test (SOAT) [49]. In brief, 5 μL of samples was placed onto the surface of MRS, TSB, or BHI (Oxoid) agar (1.5%, *w*/*v*) plates, overlaid with a soft agar (0.8%, *w*/*v*) containing fresh overnight cultures (*ca.* 10^5^ CFU/mL) of the indicators (*Lc. garvieae* CF00021, *Yersinia ruckeri* LMG3279, *Aeromonas hydrophila* CECT839, *A. salmonicida* CLFP-23, *A. salmonicida* CECT4237, *Ls. anguillarum* CECT4344, and *S. parauberis* LMG22252). The antimicrobial activity of the synthesized bacteriocins SlvV, BlpK, and BlpE was assessed in two ways, individually and in combination. When each bacteriocin was evaluated individually, 5 µL was added. When tested together, the bacteriocins (BlpK-SlvV, BlpK-BlpE, and BlpE-SlvV) were mixed at an equal ratio (1:1).

## 3. Results 

### 3.1. WGS, Assembly, and Mapping of S. salivarius MDI13 and L. sakei MEI5

The WGS of *S. salivarius* MDI13 and *L. sakei* MEI5, two interesting potential probiotic candidates previously isolated from the gut of European hakes caught in the Northeast Atlantic Ocean [50], was determined and analyzed. In this respect, both genomes were found to meet the criteria established by EFSA [22], with contig counts below 500 (29 and 15 contigs in *S. salivarius* MDI13 and *L. sakei* MEI5, respectively) (Table 3).

**Figure 1:**
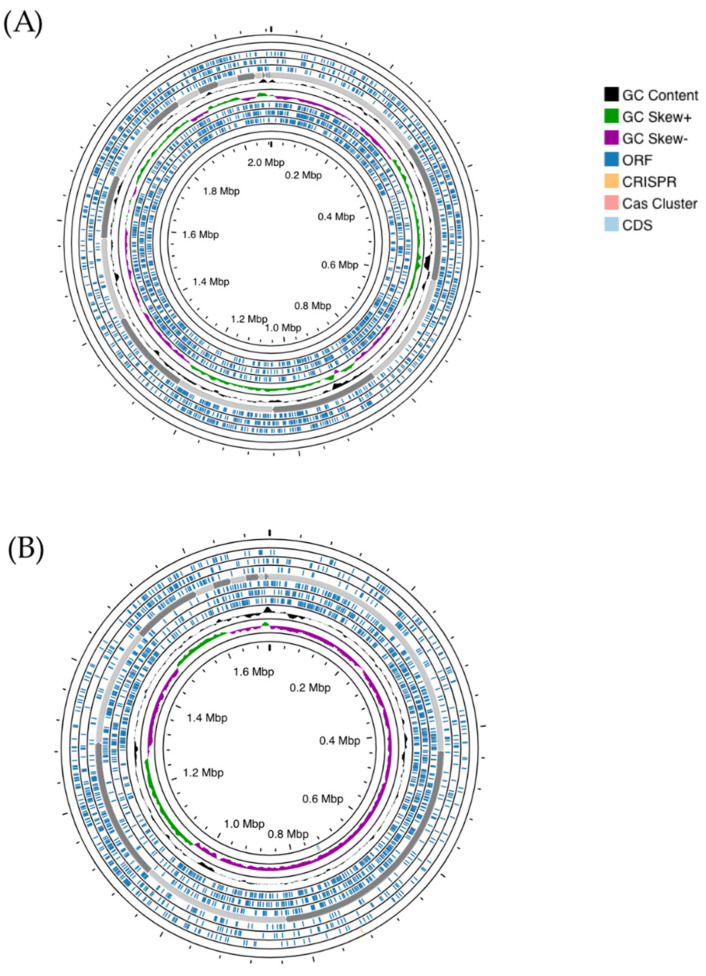
Genome mapping of *S. salivarius* MDI13 (**A**) and *L. sakei* MEI5 (**B**), generated using the *Proksee* webserver. ORFs (Open Reading Frames) and CG skew + and − are indicated in dark blue, green, and violet, respectively. CRISPR arrays and *cas* cluster systems are shown in orange and light pink, respectively.

The genome of *S. salivarius* MDI13 comprises 29 contigs (2,088,084 bp), with 40.0% of G + C content, and N50 and L50 values of 195,997 and 5, respectively. In addition, this genome contains 219 gene subsystems (set of genes that synthesize proteins with similar or associated functions). The total numbers of coding DNA sequences (CDSs) and RNAs were 1,952 and 39, respectively. On the other hand, the genome of *L. sakei* MEI15 consists of 37 contigs (1,712,091 bp), with 37.0% of G + C content, and N50 and L50 values of 237,072 and 3, respectively. Moreover, this genome contains 197 gene subsystems. The total numbers of CDSs and RNAs were 1,711 and 48, respectively (Table 3). In addition, a genomic map was made in order to visualize features of interest in the bacterial genomes (Figure 1).

### 3.2. Bioinformatic and Functional Analyses of the Genome of S. salivarius MDI13 and L. sakei MEI5

#### 3.2.1. Species Identification

In this work, we proceeded to sequence the genome of *S. salivarius* MDI13 and *L. sakei* MEI5. The *SpeciesFinder* and *KmerFinder* software programs confirmed their identification as *S. salivarius* MDI13 and *L. sakei* MEI5.

**Figure 2:**
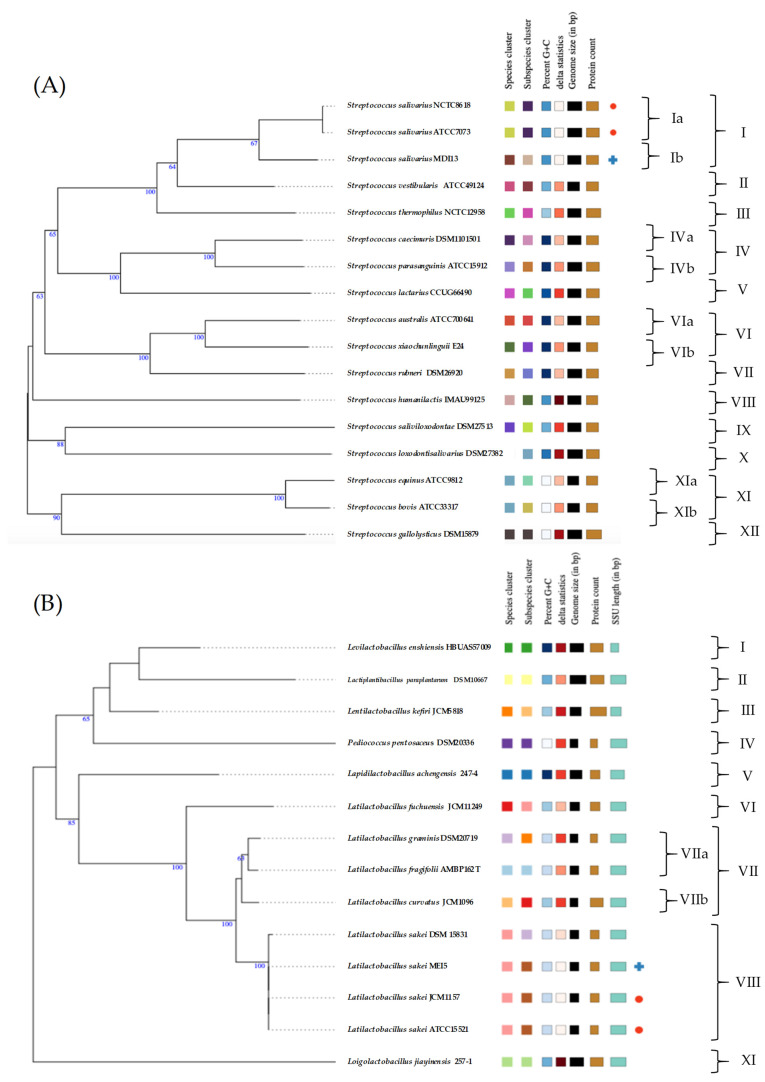
Genome-based phylogenetic tree of *S. salivarius* MDI13 (**A**) and *L. sakei* MEI5 (**B**) created by the TYGS webserver. Blue crosses indicate the probiotic candidates evaluated in this study and red dots point out the species type strains.

The TYGS server was used to perform species and subspecies similarity estimations from the closest defined type genome sequences. TYGS phylogenetic tree for the two probiotic candidates showed that *S. salivarius* MDI13 constitutes a single subcluster (Ib) with respect to subcluster Ia of the species type strains (*S. salivarius* NCTC8618 and *S. salivarius* ATCC7073; 67% identity). On the other hand, *L. sakei* MEI5 is part of a single cluster (VIII) including the species type strains (*L. sakei* JCM1157 and *L. sakei* ATCC15521; 100% identity) (Figure 2).

#### 3.2.2. Distribution of Functional Genetic Subsystems

**Figure 3:**
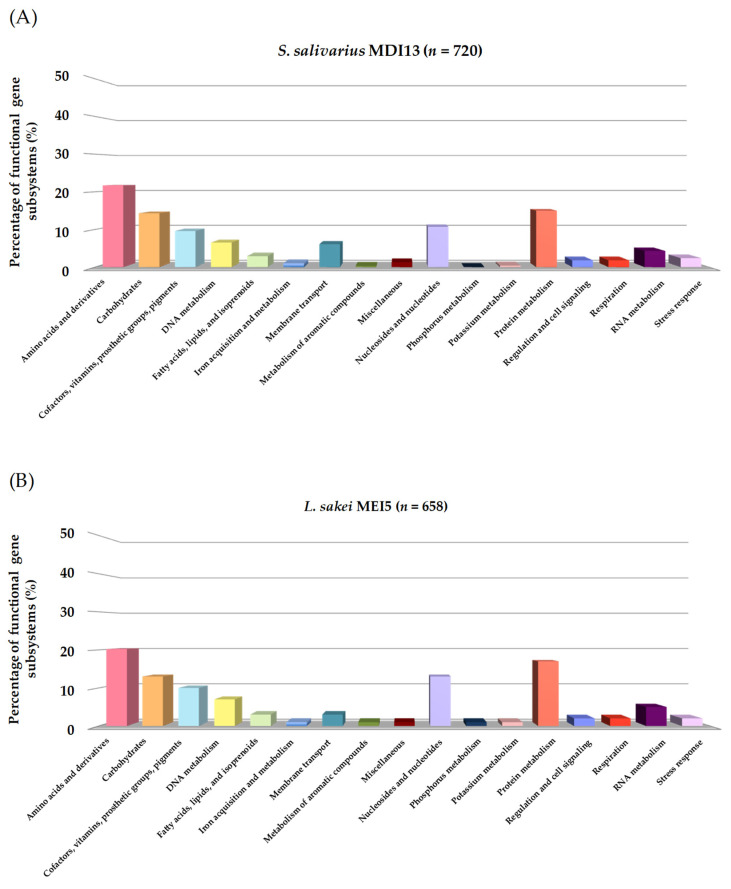
Functional gene subsystems identified in the genome of *S. salivarius* MDI13 (**A**) and *L. sakei* MEI5 (**B**), obtained using the *RAST* program.

Using the *RAST* platform, the sequences of *S. salivarius* MDI13 and *L. sakei* MEI5 were analyzed, and 17 functional gene subsystems were identified (Figure 3). *S. salivarius* MDI13 showed a high number of genes associated with the biosynthesis of amino acids and derivatives (156; 21.7%), followed by protein metabolism (108; 15.0%), carbohydrate metabolism (103; 14.3%), and nucleosides and nucleotides (77; 10.7%). In the *L. sakei* MEI5 genome, genes related to amino acids and derivatives (133; 20.2%), protein metabolism (111; 16.9%), nucleosides and nucleotides (88; 13.4%), and carbohydrate metabolism (83, 12.6%) were also identified. In both genomes, genes associated with motility and chemotaxis, nitrogen metabolism, and photosynthesis were not found.

#### 3.2.3. Identification of Genetic Determinants Related to Various Probiotic Traits

For the determination of potential probiotic characteristics, *RAST* annotation of the assembled genome of *S. salivarius* MDI13 and *L. sakei* MEI5 was used to identify genes involved in probiotic properties. In the case of the genome of *S. salivarius* MDI13, genes associated with vitamin biosynthesis, adhesion and aggregation, and amino acid metabolism were identified (Table S1). On the other hand, in the genome of *L. sakei* MEI5, not only were genes involved in adhesion and aggregation, vitamin biosynthesis, amino acid metabolism detected, but also genes responsible for antimicrobial activity, active metabolism, and stress adaptation/host gastrointestinal tract adaptation (tolerance to temperature, acid, pH, bile salts, osmotic stress, and oxidative stress) (Table S1).

The genome analyses of each strain evaluated in this work identified the existence of genes related to adhesion and aggregation. In this regard, *S. salivarius* MDI13 and *L. sakei* MEI5 had genes encoding proteins for the capacity to adhere to the host cells through the gastrointestinal tract, including those encoding the fibronectin-binding protein, exopolysaccharide (EPS), triosephosphate isomerase, and sortase A. Moreover, *L. sakei* MEI5 also had the enolase gene. 

Regarding vitamin biosynthesis, the *RAST* software predicted the existence of genes linked to the biosynthesis of vitamins, such as thiamine (B1), riboflavin (B2), pyridoxin (B6), biotin (B7), and folate (B9), in the genome of *S. salivarius* MDI13 and *L. sakei* MEI5 (Table S1). The genome analyses also allowed the detection of genes associated with amino acid biosynthesis. Specifically, *L. sakei* MEI5 encodes genes related to the biosynthesis of tryptophan, leucine, arginine, taurine, methionine, threonine, and lysine, while *S. salivarius* MDI13 only presented genes related to the biosynthesis of threonine and tryptophan (Table S1). Genes related to the production of lactic acid, such as D-lactate and L-lactate dehydrogenase, were only predicted in the genome of *L. sakei* MEI5 (Table S1). In addition, genes related to stress adaptation (e.g., tolerance to acid, pH, temperature, bile salts, and osmotic and oxidative stress) were detected in the genome of *L. sakei* MEI5 but not in *S. salivarius* MDI13 (Table S1). 

#### 3.2.4. Identification of Antibiotic Resistance Genes and Other Virulence Factors

Regarding the safety evaluation of the two probiotic candidate LAB strains characterized in this work, no genes encoding virulence factors were detected using the *VirulenceFactor 2.0* database and the *PathogenFinder* platform. In addition, no resistance genes were detected in any of the two probiotic candidates using the *ResFinder 4.1* database.

#### 3.2.5. Identification of Mobile Genetic Elements (MGEs) and CRISPR-Cas Systems

In the identification of MGEs, the possible existence of ISs was analyzed through the *ISfinder* program. ISs are DNA sequences that can move or integrate into the bacterial genome. The results revealed the presence of 7 and 11 true ISs in *S. salivarius* MDI13 and *L. sakei* MEI5 genomes, respectively (Table 4), which means that each ORF was identified as a transposase and the detected ISs displayed an e-value of 0.0 [51].

No prophage was predicted by the *Prophage Hunter* web service in *S. salivarius* MDI13 or in *L. sakei* MEI5 (Table 4). After analysis with the *PlasmidFinder* server, no plasmids were found in the genome of *S. salivarius* MDI13 and *L. sakei* MEI5 (Table 4). The *CRISPRCas-Finder* program predicted the existence of CRISPR assemblies with an evidence level of 1 and 2 in the genome of *L. sakei* MEI5, whereas none were detected in *S. salivarius* MDI13 (Table 4). 

#### 3.2.6. Identification of Operons Responsible for the Production of Bacteriocins

**Figure 4:**
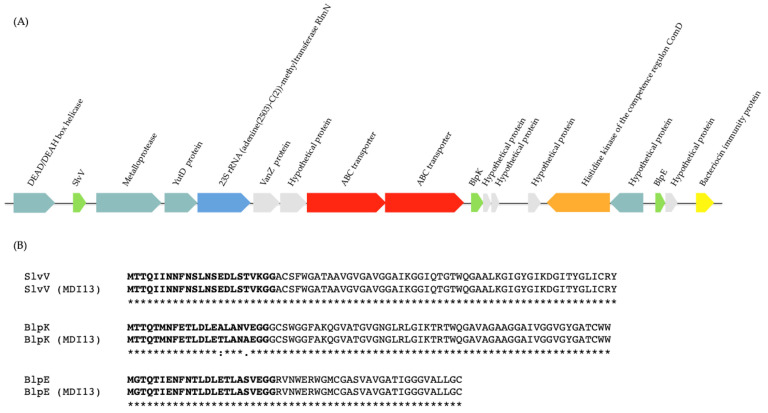
(**A**) Gene cluster encoding the bacteriocins SlvV, BlpK, and BlpE in the genome of *S. salivarius* MDI13 using *BAGEL v.4.0*. ORFs are indicated by arrows and their putative functions are indicated. (**B**) Sequence alignment of the amino acid sequence of bacteriocins (SlvV, BlpK, and BlpE) detected in the genome of *S. salivarius* MDI13, with the amino acid sequence of bacteriocins previously described in this species [59]. The leader sequences with the double glycine are shown in bold. A single fully conserved residue is designated by an asterisk (*), conserved groups of residues with robustly similar properties are illustrated with a colon (:), and groups of residues that are feebly similar are shown by a period (.).

In our study, the *BAGEL4* platform and *BLASTp* (NCBI) database were used to identify operons associated with the production of bacteriocins and to predict their functions, respectively. The bioinformatic analyses with both servers allowed us to detect in the genome of *S. salivarius* MDI13 a cluster with the structural genes of three putative bacteriocins (SlvV, BlpK, and BlpE) belonging to the class IId, taking into account the classification proposed by Álvarez-Sieiro et al. (2016) [52] (Figure 4A). In contrast, no bacteriocin genes were identified in the genome of *L. sakei* MEI5. 

The multi-bacteriocinogenic gene cluster found in the genome of *S. salivarius* MDI13 (Figure 4A) showed a genetic structure similar to other *blp* operons characterized in *S. salivarius*, *Streptococcus pneumoniae*, and *Streptococcus thermophilus*, composed of genes encoding the following: (i) putative bacteriocins with the common double glycine (2-Gly) cleavage site in the leader peptides, (ii) an ATP-Binding Cassette (ABC) transporter system, (iii) a bacteriocin immunity protein, and (iv) a protein-histidine-kinase [53,54,55,56,57,58,59]. The identity of the predicted sequences of the three bacteriocins encoded in the genome of *S. salivarius* MDI13 was compared to those of the bacteriocins previously described in this species [59], using the *Clustal Omega* server (Figure 4B). The amino acid sequences of the bacteriocins encoded by *S. salivarius* MDI13 were 100% identical to those previously described [59]. However, two amino acids of the leader peptide of BlpK in *S. salivarius* MDI13 differed from those found in other strains (Figure 4B). 

### 3.3. Evaluation of Mucin Degradation, Bile Salt Deconjugation, and Biogenic Amine Production

Neither of the two candidate probiotic LAB strains evaluated in this work showed mucinolytic activity, the capability to deconjugate bile salts, or the ability to produce biogenic amines.

### 3.4. In Vitro Cell-Free Protein Synthesis (IV-CFPS) of Bacteriocins and Evaluation of Their Antimicrobial Activity

**Figure 5:**
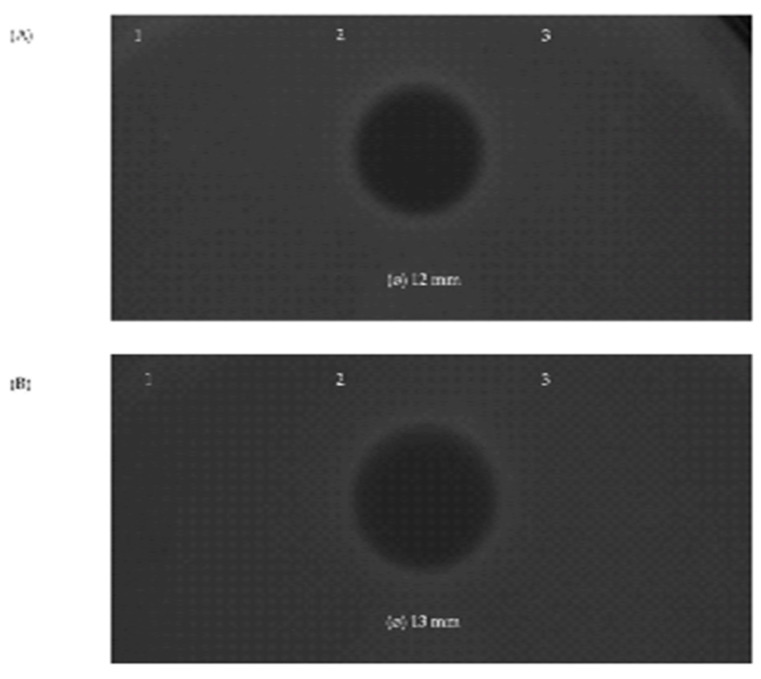
Antimicrobial activity of the in vitro-synthesized bacteriocins SlvV (1), BlpK (2), and BlpE (3) against the ictyophathogens *Lc. garvieae* CF00021 (**A**) and *S. parauberis* LMG22252 (**B**) by using a SOAT.

The three bacteriocins found to be encoded by the genome of *S. salivarius* MDI13 were synthesized by an in vitro cell-free protein synthesis (IV-CFPS) procedure and tested by a SOAT in order to evaluate their antimicrobial activity (Figure 5). In this sense, the in vitro-synthesized BlpK showed antimicrobial activity against *Lc. garvieae* CF00021 and *S. parauberis* LMG22252 (Figure 5). 

In contrast, the in vitro-synthesized SlvV and BlpE did not show antimicrobial activity against any of the evaluated pathogens. Furthermore, the combination of two peptides (BlpK-SlvV, BlpK-BlpE, and BlpE-SlvV) did not show synergistic activity; nonetheless, the inhibition zone produced by BlpK was observed in all cases. 

## 4. Discussion

The development of massive sequencing platforms together with the reduction in the cost of this technique have facilitated the sequencing of a large number of LAB genomes from different origins, which has allowed obtaining additional information on the potential biotechnological applications of these bacteria in the food industry, human medicine, veterinary field, and animal production [60,61].

The genome analysis of the strains studied, *S. salivarius* MDI13 and *L. sakei* MEI5, showed comparable annotation characteristics, with 1952 and 1711 CDSs organized in 219 and 197 subsystems, respectively. Similarly, a large proportion of the subsystems have also been predicted in other genera of LAB and assigned to amino acids and derivatives, protein metabolism, and carbohydrate metabolism [62,63].

Regarding the genetic determinants related to probiotic traits, *S. salivarius* MDI13 and *L. sakei* MEI5 had genes encoding proteins for the ability to adhere to host cells through the gastrointestinal tract, including those encoding fibronectin-binding protein, exopolysaccharide (EPS), triosephosphate isomerase, and sortase A. In addition, *L. sakei* MEI5 also had the enolase gene, which encodes a surface protein that mediates bacterial adhesion to laminin, which is a suitable attribute to be a probiotic candidate [64]. Regarding fibronectin, this is a frequent substrate for bacterial adhesins in the host gut and fibronectin-binding proteins have been recognized in many different bacteria. Because of their adhesive property, fibronectin-binding proteins from probiotic bacteria could be used for the exclusion of pathogens from the host gut and serve as a substrate for adhesion to the host epithelium and intestinal tract, which is key for the colonization and persistence of bacteria in the host intestine [65]. Other studies also detected the presence of genes encoding adhesins and sortase A in *S. salivarius* [66,67]. Moreover, several articles state that sortase A plays an important function in bacterial adhesion, biofilm formation and immunity [68,69]. Similarly, bacterial exopolysaccharides have been associated with adhesion to surfaces and protection against host immune systems [70]. Regarding vitamin biosynthesis, the *RAST* software predicted the presence of genes involved. Other studies have reported the ability of strains of *L. sakei* and *Streptococcus* spp. to produce folate, riboflavin, and thiamine [71,72,73,74]. In this regard, other studies confirm that LAB can synthesize essential biomolecules (e.g., vitamins or bioactive peptides) that can influence the composition, processing, and organoleptic properties, as well as the overall quality of, food and feed. In addition, they can enhance synergistic effects on digestion and alleviate symptoms of intestinal malabsorption [75,76]. It has been suggested that dietary micronutrients, including B vitamins, may impact fillet texture and increase the number of muscle cells in post-smolts [77]. In addition, the synthesis of B group vitamins, specifically thiamine (vitamin B_1_), could reduce the numbers of dead and deformed fry in the probiotic-diet-fed fish [51,78].

The genome analyses also allowed the identification of genes related to amino acid biosynthesis (tryptophan, leucine, arginine, taurine, methionine, threonine, and lysine). In this sense, tryptophan is a metabolic precursor of serotonin, melatonin, and niacin (vitamin B3) which is involved in numerous physiological functions, including the modulation of behavior, antioxidant, and immune and stress responses. Aquaculture has spent years optimizing the amount of tryptophan in feed, as this is an essential aromatic amino acid that must be supplied through the fish feed [79]. In the case of leucine, this is a branched-chain amino acid, nutritionally essential in animals. This is one of the most abundant amino acids in high-quality protein feeds and is involved in energy metabolism (i.e., glucose uptake and mitochondrial biogenesis), thus providing energy for protein synthesis [80]. In this regard, approximately 80% of leucine is used for protein synthesis, [80]. Zhao et al. (2023) [81] suggested that fish feed with a diet supplemented with leucine could recover the function of the intestinal barrier by increasing antioxidant capacities, humoral immune response, and the level of tight junction protein. On the other hand, among the essential amino acids, arginine is one of the functional amino acids in animals. Specifically, arginine is an indispensable amino acid for fish, but its endogenous biosynthesis is limited. In particular, arginine specifically modulates the adenosine 5′-monophosphate (AMP)-activated protein kinase (AMPK) pathway to regulate energy balance and also activates the target of the rapamycin (TOR) signaling pathway to regulate protein synthesis [82,83]. Cysteine is a precursor of taurine, and has physiological roles, such as antioxidant stress and immune enhancement. Studies have shown that cysteine supplementation greatly increases the weight and growth rate of juvenile golden pompano (*Trachinotus ovatus*) [84]. Furthermore, the possibility of the reduction in mercury contamination in fish with dietary cysteine supplementation has been proposed [85]. Methionine is also an essential amino acid that stimulates protein synthesis, reduces green liver syndrome, increases cell survival, and improves digestibility and growth in some fish species, for instance rainbow trout (*Oncorhynchus mykiss*) and Atlantic salmon (*Salmo salar*) [86,87].

Genes involved in the production of lactic acid and related to stress adaptation were predicted in the genome of *L. sakei* MEI5. In this sense, one of the beneficial properties of lactic acid production is to hinder the survival of pathogenic bacteria [51,88,89,90]. The bacterial tolerance to acid and bile salts represents an adaptive advantage for probiotics, as it allows them to survive during their transit through the stomach and small intestine and, therefore, to reach the large intestine in greater numbers and with greater viability [91,92].

Regarding the safety evaluation of the two probiotic candidate LAB strains characterized in this work, no genes encoding virulence factors were detected. Although some *S. salivarius* strains have been used as oral probiotics [93], a recent EFSA report has notified that this species is not recommended for the QPS list because of its potential pathogenic characteristics [15]. However, as mentioned above, bioinformatic analyses of the *S. salivarius* MDI13 genome did not identify any virulence factor. Regarding *L. sakei*, this species is considered potentially as safe as many other LAB species with the QPS status [94]. Independently of this, all strains proposed as probiotics for animals and humans should be evaluated for susceptibility to antibiotics of relevance in therapeutic practice in humans and animals [94]. In this regard, the identification of acquired transmissible antibiotic resistance(s) in a strain would automatically invalidate its use, due to the risk of spreading the resistance genes to commensal and pathogenic bacteria, which would increase the current major public health problem generated by the emergence and dissemination of antibiotic (multi)resistant bacteria [16]. In our study, no resistance genes were detected in any of the two probiotic candidates using the *ResFinder 4.1* database. These results are in agreement with our previous in vitro assays in which both strains were found to be sensitive to the most commonly used antibiotics in human medicine and animal health (unpublished data).

Concerning the identification of MGEs, no prophage was detected in *S. salivarius* MDI13 or *L. sakei* MEI5. This result can be considered as a positive probiotic characteristic, since some prophages may be implicated in antibiotic resistance and/or in the development of virulent characteristics [36,95]. Also, no plasmids were found in the genome of *S. salivarius* MDI13 and *L. sakei* MEI5. On the other hand, CRISPR assemblies (evidence level of 1 and 2) were found in the genome of *L. sakei* MEI5. In this regard, sequences with an evidence level lower than 3 were discarded as they indicate potentially invalid CRISPR assemblies [38,96]. Furthermore, the absence of associated *cas* genes in the predicted CRISPR arrays in the *L. sakei* MEI5 genome suggests the presence of orphan loci. This finding is not a surprising feature, as they have been previously described in other bacteria as remnants of previous functional CRISPR-Cas systems [97,98].

On the other hand, the strains studied do not have mucinolytic capacity. The gastrointestinal epithelium is covered by a mucus layer that prevents the penetration of microorganisms, so bacteria with mucinolytic capacity are considered pathogenic for fish by facilitating their extraintestinal migration [11,99]. According to the FAO, the capacity to deconjugate bile salts is an advantageous characteristic in probiotics for use in humans [6]. This beneficial effect was attributed to the correlation observed amongst the capability of bacteria to deconjugate bile salts with the capability to survive in the gastrointestinal tract [100]. Although this finding was supported by later studies [101,102], there are some studies that found no relationship between survival in the presence of bile and the ability to deconjugate bile salts [103,104]. In any case, the deconjugation of bile salts makes them less effective at emulsifying ingested lipids, thereby reducing fatty acid absorption and the reabsorption of bile acids themselves [105]. Although this may be a desirable feature in human nutrition, it is not desirable in production animals, as their feed becomes less efficient. Furthermore, according to some authors, the deconjugation of bile salts can lead to the formation of toxic compounds that alter the intestinal microbiota, resulting in diarrhea, enteritis, or the activation of carcinogens in the intestinal contents [11,51,106,107,108]. Moreover, none of the strains evaluated in this work showed the capacity to produce biogenic amines, which are organic bases of low molecular size found in various foods that can exert adverse effects with symptoms similar to allergy [43]. Histamine and tyramine have been extensively studied because of their toxic effects due to their vasoactive and psychoactive properties. In this regard, there is legislation in the European Union that regulates the presence of histamine in fish [109]. Tyramine is related to migraines and hypertensive crises in patients treated with the enzyme monoamine oxidase inhibitor. Regarding putrescine and cadaverine, the problem lies in the fact that they potentiate the effects of other biogenic amines and/or hinder their detoxification in the body [51,110,111,112,113].

The bioinformatic analyses allowed the detection of a cluster with the structural genes of three putative bacteriocins (SlvV, BlpK, and BlpE) in the genome of *S. salivarius* MDI13. Previous studies focused on genome mining from marine bacteria have also predicted bacteriocin gene clusters [114], including *blp* operons [115]. Interestingly, previous studies reported that approximately 50% of the salivaricins or related peptides are unique to *S. salivarius*, whereas others (e.g., BlpK) are mostly identified in other streptococcal species (e.g., *S. thermophilus*, *S. pneumoniae*, and *S. pyogenes*) [59]. In recent decades, a huge number of bacteriocins produced by LAB have been described [48,51,61,116]. In this regard, bacteriocin production by LAB is considered an important probiotic antimicrobial strategy to inhibit the growth of other sensitive strains co-existing in the same ecosystem, colonizing their microbial community and displacing pathogens [115], and is also linked to immunostimulatory effects [117]. In our work, BlpK synthesized in vitro showed antimicrobial activity against *Lc. garvieae* CF00021 and *S. parauberis* LMG22252. In another study, BlpK produced in vitro by a cell-free system was shown to be active against *S. salivarius*, *S. thermophilus*, and *Lactococcus lactis* [59]. In addition, a recombinant strain encoding BlpK exerted antimicrobial activity against phylogenetically closely related streptococcal species, and non-closely related pathogenic species, such as *Listeria monocytogenes*, *Enterococcus faecium*, *Enterococcus faecalis*, and *Staphylococcus aureus* [59]. To our knowledge, our study reports for the first time the antimicrobial activity of this broad-antimicrobial-spectrum bacteriocin against the fish pathogens *Lc. garvieae* and *S. parauberis*. Nevertheless, taking into account that the production of most bacteriocins in *Streptococcus* spp. is induced by transcriptional regulatory systems that require in most cases that the producer microorganism grows in presence of the indicator microorganism, future works will be needed to demonstrate that this bacteriocin is produced by *S. salivarius* MDI13 in liquid coculture with the microorganism of interest, followed by bacteriocin purification and detection by mass spectrometry. On the other hand, SlvV and BlpE did not exert antimicrobial activity against any of the evaluated pathogens. Furthermore, the combination of two peptides (BlpK-SlvV, BlpK-BlpE, and BlpE-SlvV) did not show synergistic activity. In this respect, it has been reported that the activity of fusion constructions between the expression–secretion signal of the *blpK* gene and the mature part of *slvV* and *blpE* in *S. salivarius* showed a narrow spectrum, targeting some streptococcal species, such as *S. thermophilus* and *S. salivarius* in the case of the expression of SlvV, and also *Streptococcus vestibularis* and *Streptococcus pyogenes* when expressing BlpE [59]. Therefore, the absence of the antimicrobial activity of SlvV and BlpE against the indicators tested in our study suggests that these microorganisms are not sensitive to both peptides.

The results obtained in this study suggest the safety and possible probiotic potential of both strains. Nevertheless, further in vivo rearing trials and challenge tests carried out in fish are needed to validate these findings.

## 5. Conclusions

This work highlights the importance of subjecting probiotic candidates for the food chain to WGS and bioinformatic and functional analyses. The bioinformatic analyses of the genomes of *S. salivarius* MDI13 and *L. sakei* MEI5, both strains isolated from European hakes, allowed confirming their safety and identifying genes related to their potential probiotic characteristics, including genes associated with vitamin biosynthesis adhesion and aggregation and amino acid metabolism. Specifically, in the case of *L. sakei* MEI5, other genes potentially related to probiotic traits were identified, such as genes related to lactic acid production, active metabolism, and adaptation to stress and adverse conditions in the host gastrointestinal tract. Moreover, in vitro-synthesized BlpK encoded by the genome of *S. salivarius* MDI13 displayed antimicrobial activity against *Lc. garvieae* and *S. parauberis*, two ichthyopathogens of utmost relevance in aquaculture. The broad antimicrobial spectrum against ichthyopathogens, the interesting probiotic properties, and the safety profile of *S. salivarius* MDI13 and *L. sakei* MEI5 reveals their potential as probiotics to prevent fish bacterial diseases in aquaculture as a complementary/alternative strategy to the use of antibiotics. However, before proposing their use as probiotics, a thorough in vivo evaluation of their safety and probiotic efficacy should be carried out. These findings are very promising, since aquaculture needs to have effective, safe, sustainable, and economically profitable preventive and therapeutic strategies for ichthyopathologies of relevance that will improve the health and productivity of aquaculture species and the safety of marketed products.

## Figures and Tables

**Table 1:** Bioinformatic tools used for genome analyses of *S. salivarius* MDI13 and *L. sakei* MEI5.

Analysis Type	Web Server Program	Website	Accessed on	Reference
Bacterial identification	*SpeciesFinder v.2.0* *KmerFinder v.3.0.2*	https://cge.cbs.dtu.dk/services/SpeciesFinder/ https://cge.cbs.dtu.dk/services/KmerFinder/	1 June 2023	Larsen et al. (2014) [27] Hasman et al. (2014) [28]
Genome annotation	*Rapid Annotations using Subsystems Technology (RAST)*	https://rast.nmpdr.org/rast.cgi	8 June 2023	Overbeek et al. (2014) [25]
Bacteriocin production	*Bagel4*	http://bagel4.molgenrug.nl/index.php	15 September 2023	Van Heel et al. (2018) [29]
Alignment between amino acid sequences	*BLAST protein*	https://blast.ncbi.nlm.nih.gov/Blast.cgi?PAGE=Proteins	15 September 2023	Singh et al. (2016) [30]
Multiple sequence alignment	*Clustal Omega*	https://www.ebi.ac.uk/jdispatcher/msa/clustalo	15 September 2023	Sievers et al. (2011) [31]
Virulence factors	*VirulenceFinder v.2.0*	http://www.mgc.ac.cn/VFs/main.htm	16 July 2023	Joensen et al. (2014) [32]
Antibiotic resistance	*ResFinder v.4.1*	https://cge.cbs.dtu.dk/services/ResFinder/	16 July 2023	Bortolaia et al. (2020) [33]
Pathogenicity	*PathogenFinder v.1.1*	https://cge.cbs.dtu.dk/services/PathogenFinder/	16 July 2023	Consentino et al. (2013) [34]
IS	*MGE*	https://cge.cbs.dtu.dk/services/MobileElementFinder/	3 September 2023	Siguier et al. (2006) [35]
Active prophages	*Prophage Hunter*	https://pro-hunter.genomics.cn/index.php/Home/hunter/hunter.html	7 September 2023	Song et al. (2019) [36]
Plasmids	*PlasmidFinder v.2.1*	https://cge.cbs.dtu.dk/services/PlasmidFinder/	7 September 2023	Carattoli et al. (2014) [37]
CRISPR-Cas system	*CRISPRCasFinder*	https://crisprcas.i2bc.paris-saclay.fr/CrisprCasFinder/Index	9 September 2023	Couvin et al. (2018) [38]
Type (Strain) Genome Server	*TYGS*	https://tygs.dsmz.de	13 September 2023	Meier et al. (2019) [39]
Genomic maps	*Proksee web server*	https://www.ebi.ac.uk/Tools/msa/clustalo/	13 September 2023	Grant et al. (2023) [26]

**Table 2:** Oligonucleotides used in this work.

Oligonucleotide Primers	Nucleotide Sequence (5′-3′) ^a^	PCR-Amplified Gene Fragment ^b^
slvV-F	**GGGAATTAATACGACTCACTATAGGGCTTAAGTATAAGGAGGAAAAAAT**ATGGCATGTAGTTTTTGGGGAGCTACAGCTGC	*slvV*
slvV-R	AAACCCCTCCGTTTAGAGAGGGGTTATGCTAGTTAATAACGACAAATAAGTCCATAAG	
blpK-F	**GGGAATTAATACGACTCACTATAGGGCTTAAGTATAAGGAGGAAAAAAT**ATGGGATGTAGCTGGGGAGGTTTTGCTAAAC	*blpK*
blpK-R	AAACCCCTCCGTTTAGAGAGGGGTTATGCTAGTTACCACCAGCATGTTGCTCCATAACC	
blpE-F	**GGGAATTAATACGACTCACTATAGGGCTTAAGTATAAGGAGGAAAAAAT**ATGCGAGTCAATTGGGAACGATGGGGAATG	*blpE*
blpE-R	AAACCCCTCCGTTTAGAGAGGGGTTATGCTAGTTAACAGCCAAGTAAGGCTACTCCACC	

^a^ T7 promoter is shown in bold; T7 transcription terminator is underlined. ^b^ Each PCR-amplified gene fragment contains the mature sequence of the bacteriocin flanked by the T7 promoter and transcription terminator.

**Table 3:** General characteristics of the assembled genome of the two sequenced LAB strains.

Characteristic	*S. salivarius* MDI13	*L. sakei* MEI5
Size (bp)	2,088,084	1,712,091
Content G + C (%) ^a^	40.0	37.0
L50 ^b^	5	3
N50 ^c^ (bp)	195,997	237,072
Contigs (nº)	29	15
Subsystems (nº)	219	197
Coding DNA sequences (nº)	1952	1711
RNA sequences (nº)	39	48

^a^ G + C content: guanine and cytosine content in each genome; ^b^ L50: smallest number of contigs whose length is half the size of the genome; ^c^ N50: length of the smallest contig of the group that constitutes 50% of the genome.

**Table 4:** Mobile genetic elements (ISs, active prophages, and plasmids) and CRISPR-Cas systems identified in the genome of *S. salivarius* MDI13 and *L. sakei* MEI5.

Analyzed MGE	*S. salivarius* MDI13	*L. sakei* MEI5
**Insertion sequences (ISs)**	**IS similar/family/bacterial species/length (bp)**	**IS similar/family/bacterial species/length (bp)**
	ISLgar4/IS6/*Lc. garvieae*/660 ISSth2/IS1182/*S. salivarius*/1897 IS1193D/ISL3/*Streptococcus thermophilus*/1675 IS1193/ISL3/*S. thermophilus*/1675 ISSmu2/ISL3/*Streptococcus mutans*/1453 ISSth8/ISL3/*S. thermophilus*/678 ISStrs1/IS200/*S. salivarius*/942	IS1310/IS256/*Entetococcus hirae*/2216 ISLpl3/IS427/*Lactobacillus plantarum*/872 IS1216E/IS6/*Entetococcus faecium*/1094 ISS1W/IS6/*Lactococcus lactis*/1086 IS1216V/IS6/*E. hirae*/1023 IS1520/IS3/*L. sakei*/2288 ISLpl1/IS30/*L. plantarum*/2020 ISPp1/IS30/*Pediococcus pentosaceus*/1901 ISLsa1/IS30/*L. sakei*/1110 ISLsa1/IS30/*L. sakei*/938
**Active prophages**	**Similar prophage/contig/length**	**Similar prophage/contig/length**
	ND ^a^	ND ^a^
**Plasmids**	**Plasmid replication origin/length**	**Plasmid replication origin/length**
	ND ^a^	ND ^a^
**CRISPR-Cas system**	**CRISPR spacers/*cas* genes/contig**	**CRISPR spacers/*cas* genes/contig/level evidence**
	ND ^a^	7/ND/13/2 1/ND/20/1 1/ND/33/1

^a^ ND: not detected.

## Data Availability

The whole-genome sequences of *S. salivarius* MDI13 and *L. sakei* MEI5 are deposited in GenBank under the accession numbers JAYKZL000000000 and JBEJGS000000000, respectively.

## Supplementary Materials

The following supporting information can be downloaded at: https://www.mdpi.com/article/10.3390/vetsci11080365/s1. Table S1. Probiotic characteristics based on genome analysis.
