# Supplementary material for: Genomic Sequence of Streptococcus salivarius MDI13 and Latilactobacillus sakei MEI5: Two Promising Probiotic Strains Isolated from European Hakes (Merluccius merluccius, L.)
Source: Vet Sci. 2024 Aug 10;11(8):365. doi: 10.3390/vetsci11080365 (PMC11359882; doi:10.3390/vetsci11080365)
Supplement: Supplementary file 1 [file vetsci-11-00365-s001.zip › vetsci-3144572-supplementary.pdf]

Table S1. Probiotic characteristics based on genome analysis[a].

Gene function	*S. salivarius* MDI13 (contig)	*L. sakei* MEI5 (contig)
**Adhesion and aggregation**		
Enolase	-	1
Fibronectin-binding protein	3	1
Exopolysaccharides (EPS) biosynthetic gene clusters	3	1
a. Exopolysaccharide biosynthesis protein	-	1
b. Glycosyl transferase, group 1 family protein	-	6
c. Manganese-dependent protein-tyrosine phosphatase	-	4
d. Tyrosine-protein kinase EpsD	3	-
e. Tyrosine-protein kinase transmembrane modulator EpsC	3	-
Triosephosphate isomerase	5	1
AntiadhesinPls	-	-
Sortase A, LPXTG	7	2
ATP synthase epsilon chain	-	-
**Vitamin biosynthesis**		
**Thiamine**		
Cytosine/purine/uracil/thiamine/allantoin permease family protein	-	-
HMP-PP hydrolase (pyridoxal phosphatase) Cof, detected in genetic screen for thiamin metabolic genes	-	-
Hydroxyethylthiazole kinase	-	3
Hydroxymethylpyrimidine ABC transporter, ATPase component	-	-
Hydroxymethylpyrimidine ABC transporter, substrate-binding component	-	-
Hydroxymethylpyrimidine ABC transporter, transmembrane component	-	-
Substrate-specific component ThiT of thiamin ECF transporter	-	-
Substrate-specific component ThiW of predicted thiazole ECF transporter	-	2
Thiamin pyrophosphokinase	2	2
Thiaminase II involved in salvage of thiamin pyrimidine moiety	-	1
Thiamine-phosphate pyrophosphorylase tRNA S (4) U 4-thiouridine synthase (formerly ThiI)	-	-
Xanthine/uracil/thiamine/ascorbate permease family protein	9	2
**Riboflavin**		
3,4-dihydroxy-2-butanone 4-phosphate synthase	-	-
6,7-dimethyl-8-ribityllumazine synthase	-	-
ATP phosphoribosyltransferase	1	1
Diacylglycerol kinase	1	2
Diaminohydroxyphosphoribosylaminopyrimidine deaminase/5- amino-6-(5-phosphoribosylamino)uracil reductase	-	-
FIG000859: hypothetical protein YebC	-	-
NADH dehydrogenase	-	1
N-terminal domain of CinA protein, COG1058/C-terminal domain of CinA type S	-	-
Orotidine 5′-phosphate decarboxylase	-	4
Phosphoribosyl-AMP cyclohydrolase/Phosphoribosyl-ATP pyrophosphatase	-	1
Riboflavin kinase/FMN adenylyltransferase	-	-
Riboflavin synthase eubacterial/eukaryotic	-	-
Ribulose-phosphate 3-epimerase	-	2
Substrate-specific component RibU of riboflavin ECF transporter	-	3
Transcription termination protein NusB	-	1
tRNA pseudouridine synthase B	-	1
**Pyridoxin**		

D-3-phosphoglycerate dehydrogenase	6	2
Hypothetical NagD-like phosphatase	-	6
1-deoxy-D-xylulose 5-phosphate synthase	-	-
Pyridoxamine 5′-phosphate oxidase	-	-
Pyridoxine biosynthesis glutamine amidotransferase, glutaminase subunit	-	-
NAD-dependent glyceraldehyde-3-phosphate dehydrogenase	-	5
**Biotin**		
3-ketoacyl-CoA thiolase Acetyl-CoA acetyltransferase	-	-
Acetoacetyl-CoA synthetase/Long-chain-fatty-acid–CoA ligase	-	-
Adenosylmethionine-8-amino-7-oxononanoate aminotransferase	-	-
ATPase component BioM of energizing module of biotin ECF transporter	-	1
Biotin synthase	14	2
Biotin—protein ligase	-	-
Biotin operon repressor	-	6
Competence protein F homolog, phosphoribosyltransferase domain	-	-
Long-chain-fatty-acid–CoA ligase	-	-
Substrate-specific component BioY of biotin ECF transporter	1	6
Biotin carboxyl carrier protein of acetyl-CoA carboxylase	5	5
Biotin carboxyl carrier protein of methylcrotonyl-CoA carboxylase	-	-
Biotin carboxylase of acetyl-CoA carboxylase	5	5
Biotin carboxylase of methylcrotonyl-CoA carboxylase	-	-
**Folate**		
Dihydrofolate synthase Folylpolyglutamate synthase	6	4
GTP cyclohydrolase I	6	4
Pantoate—beta-alanine ligase	-	-
Aspartate 1-decarboxylase	-	-
2-amino-4-hydroxy-6-hydroxymethyldihydropteridine	6	-
Dihydroneopterin aldolase	6	-
Dihydropteroate synthase	-	-
Cell division protein FtsH	-	5
Hypoxanthine-guanine phosphoribosyltransferase tRNA(Ile)-lysidine synthetase	-	1
ARNt (Ile)-lisidine synthetase	-	-
Para-aminobenzoate synthase, amidotransferase component	2	-
5-formyltetrahydrofolate cyclo-ligase	4	2
Timidilato synthase	-	-
Dihydrofolate reductase	1	2
**Aminoacids metabolism**		
**Threonine**		
Biosynthetic aromatic amino acid aminotransferase alpha	-	-
Aspartokinase	5	2
Homoserine dehydrogenase	5	2
Aspartate aminotransferase	3	2
Aspartate-semialdehyde dehydrogenase	-	6
Homoserine kinase	5	2
Threonine synthase	-	2
**Tryptophan**		
Tryptophan synthase alpha chain	-	1
Tryptophan synthase beta chain	-	-
Phosphoribosylformimino-5-aminoimidazole carboxamide ribotide isomerase	1	1

Para-aminobenzoate synthase, amidotransferase component	-	-
Isochorismatase	-	4
Indole-3-glycerol phosphate synthase	2	4
Anthranilate phosphoribosyltransferase	2	4
Anthranilate synthase, amidotransferase component	2	4
**Methionine**		
Cystathionine beta-lyase	-	-
Cystathionine gamma-synthase	-	-
S-adenosylmethionine synthetase	-	1
Serine acetyltransferase	-	2
Homoserine O-succinyltransferase (EC 2.3.1.46)	-	1
Methionine ABC transporter ATP-binding protein	-	1
5-methyltetrahydrofolate—homocysteine methyltransferase	-	-
5,10-methylenetetrahydrofolate reductase	-	2
O-acetylhomoserine sulfhydrylase	-	1
SAM-dependent methlytransferase YrrT	-	-
**Leucine**		
3-isopropylmalate dehydrogenase	-	1
3-isopropylmalate dehydratase small subunit	-	1
2-isopropylmalate synthase	-	1
**Lysine**		
Aspartokinase	-	2
Lysine-epsilon oxidase	-	-
4-hydroxy-tetrahydrodipicolinate synthase	-	1
4-hydroxy-tetrahydrodipicolinate reductase	-	1
Aspartate-semialdehyde dehydrogenase	-	6
Diaminopimelate decarboxylase	-	1
N-acetyl-L,L-diaminopimelate deacetylase	-	1
2,3,4,5-tetrahydropyridine-2,6-dicarboxylate N-acetyltransferase	-	1
N-acetyl-L,L-diaminopimelate aminotransferase	-	-
**Cysteine**		
Cysteine synthase	-	-
Phosphoadenylyl-sulfate reductase [thioredoxin]	-	-
Cysteinyl-tRNA synthetase	-	6
Serine acetyltransferase	-	2
Sulfite reductase [NADPH] hemoprotein beta-component	-	-
Sulfite reductase [NADPH] flavoprotein alpha-component	-	-
Phosphoribosyl-AMP cyclohydrolase	-	1
Imidazole glycerol phosphate synthase cyclase subunit	-	1
Imidazoleglycerol-phosphate dehydratase	-	1
Histidinol dehydrogenase	-	1
ATP phosphoribosyltransferase	-	1
Histidinol-phosphate aminotransferase	-	1
**Arginine**		
N-acetyl-gamma-glutamyl-phosphate reductase	-	-
N-acetylglutamate synthase	-	-
Acetylglutamate kinase	-	3
Acetylornithine aminotransferase	-	3
Ornithine carbamoyltransferase	-	1
Argininosuccinate synthase	-	1

Argininosuccinate lyase	-	1
Arginine pathway regulatory protein ArgR	-	2
**Production of lactic acid**		-
D-lactate dehydrogenase	-	3
L-lactate dehydrogenase	-	3
**Active metabolism**		
Poly (glycerol-phosphate) alpha-glucosyltransferase	-	6
Beta-1, 3-glucosyltransferase	-	-
Xylose isomerase domain protein TIM barrel	-	-
**Enzyme production for food digestion**		
Amylase	-	-
Lipase	-	-
Serina proteasa extracelular	-	-
Phytase	-	-
Cellulase	-	-
Xylanase	-	-
**Stress adaptation / host gastrointestinal tract adaptation**		
**Temperature tolerance**		1
Cold shock protein CspA	-	1
Cold shock protein CspC	-	1
Cold shock protein CspP	-	1
Heat shock protein DnaJ	-	-
Heat shock protein DnaK	-	-
Heat shock protein HtpX	-	2
Heat shock protein Hsp33	-	-
Heat shock protein GrpE	-	4
Heat shock protei Hsp70	-	-
Small heat shock protein	-	-
Ribosome-associated heat shock protein	-	1
Co-chaperonin GroES (heat shock protein)	-	-
Molecular chaperone GroEL (heat shock protein)	-	-
**Acid tolerance**		
ATP synthase subunit aa	-	6
ATP synthase subunit b	-	6
ATP synthase subunit c	-	6
ATP synthase alpha chain a	-	6
ATP synthase Beta chain	-	6
ATP synthase Gamma chain	-	6
ATP synthase Epsilon chain	-	6
ATP synthase delta chain	-	6
ATP-dependent Clp protease ATP-binding subunit	-	1
Glucose-6-phosphate isomerase	-	5
GTP pyrophosphokinase	-	1
Pyruvate kinase	-	2
**pH**		
Sodium-proton antiporters	-	-
Alkaline shock proteins	-	1
F0F1-ATPase	-	-
**Bile salts tolerance**		
Choloylglycine hydrolase	-	-

Bile salt hydrolase	-	-
Glucosamine-6-phosphate deaminase	-	5
CTP synthase	-	1
DamX	-	-
**Osmotic stress tolerance**		
Glycine betaine/carnitine/choline	-	-
ABC transporter, ATP-binding protein stress	-	1
ABC transporter, substrate binding and permease	-	1
ABC transporter, permease protein	-	1
Transport protein	-	-
**Oxidative stress tolerance**		
Catalase	-	-
Thiol peroxidase	-	3
Glutathione reductase	-	-
Glutathione peroxidase	-	4
NADH peroxidase	-	10
NADH oxidase	-	-
NADH flavin oxidoreductase	-	-
Halo peroxidase	-	-
Iron dependent peroxidase	-	-
Thioredoxin	-	2
Thioredoxin reductase (NADPH)	-	5
Pyruvate oxidase	-	10
Dihydroorotate oxidase	-	-
Cadmium-/manganese-transporting P-type ATPase	-	-
Manganese ABC transporter	-	5
**Short-chain fatty acids**		
Succinate dehydrogenase	-	-
Fumarate reductase	-	-
Butyrate kinase	-	-
Butyryl-CoA:acetate-CoA transferase	-	-
Butyryl-CoA dehydrogenase electron-transferring flavoprotein complex	-	-
Butyryl transferase	-	-
**Inmunomodulation**		
D-lactate dehydrogenase	-	3
D-alanine poly (phosphoribitol) ligase	-	1
Exopolysaccharides (EPS) biosynthetic gene clusters	3	1
a. Exopolysaccharide biosynthesis protein	-	1
b. Glycosyl transferase, group 1 family protein	-	6
c. Manganese-dependent protein-tyrosine phosphatase	-	4
d. Tyrosine-protein kinase EpsD	3	-
e. Tyrosine-protein kinase transmembrane modulator EpsC	3	-
Von Willebrand factor A-type domain	-	-
**Modulation of neurotransmisor**		
Poly-gamma-glutamate synthesis protein	-	-
Enzimas GABA	-	-
Glutaminc acid decarboxylase	-	-
2 oxoglutarate dehydrogenase complex	-	-
GABA amino transferase	-	-
glutamate/GABA antiporter GadC	-	-

glutamic acid	-	-
pyridoxal 5'-phosphate cofactor	-	-
**Production of mucin**		
Succinate dehydrogenase	-	-
Fumarate reductase	-	-
Butyrate kinase	-	-
Butyryl-CoA:acetate-CoA transferase	-	-
Butyryl-CoA dehydrogenase electron-transferring flavoprotein complex	-	-
Butyryl transferase	-	-

[a]-: not detected.
